# A small collection of *Endeis* juveniles (Arthropoda: Pycnogonida: Endeidae) sorted from the fouling organisms on the R/V Dayangyihao

**DOI:** 10.3897/BDJ.9.e62343

**Published:** 2021-04-08

**Authors:** Jianjia Wang, Dong Sun, Peng Tian, Dinyong Huang, Wentao Niu, Feng Zhang

**Affiliations:** 1 Third Institute of Oceanography, Ministry of Natural Resources, Xiamen, China Third Institute of Oceanography, Ministry of Natural Resources Xiamen China; 2 The Key Laboratory of Invertebrate Systematics and Application, College of Life Sciences, Hebei University, Baoding, China The Key Laboratory of Invertebrate Systematics and Application, College of Life Sciences, Hebei University Baoding China; 3 Key Laboratory of Marine Ecosystem Dynamics, Second Institute of Oceanography, Ministry of Natural Resources, Hangzhou, China Key Laboratory of Marine Ecosystem Dynamics, Second Institute of Oceanography, Ministry of Natural Resources Hangzhou China

**Keywords:** Indian Ocean, Mauritius, new record, fouling communities on ship hulls

## Abstract

**Background:**

*Endeis straughani* Clark, 1970 was originally described from Queensland, Australia. Its range was extended to Ghana, West Africa when *E. picta* Bamber, 1979 was synonymised with *E. straughani* by Staples (1982). The current paper extends this range further, to include Port Louis, Mauritius.

**New information:**

Five immature individuals gathered amongst hydroids during the 5^th^ leg of the DY125-34 expedition were tentatively identified as *E. straughani* juveniles. Since these were collected from the fouling community on the ship’s hull, they probably originated in Port Louis (Mauritius) when the ship was docked there.

## Introduction

The genus *Endeis* Philippi, 1843 includes 18 species distributed widely, except from the Arctic Ocean (Takahashi et al. 2007, Bamber et al. 2020). Stock (1968) established a key to 13 species, while Fry and Hedgpeth (1969) set up a key to seven species and there were also some regional keys for those species occurring in South Africa (Barnard 1954), Middle America (Child 1979), Japan (Nakamura and Child 1983), Colombia Caribbean Coast (Müller and Krapp 2009) and north-east Atlantic (Bamber 2010). After Stock’s key, seven *Endeis* species were described by Stock (1970), Clark (1970), Pushkin (1976), Bamber (Bamber 1979, Bamber 1992), Takahashi et al. (2007) and Müller and Krapp (2009), respectively, but *Endeis picta* Bamber, 1979 was synonymised with *Endeis straughani* Clark, 1970 (Staples 1982).

**Figure 1.**
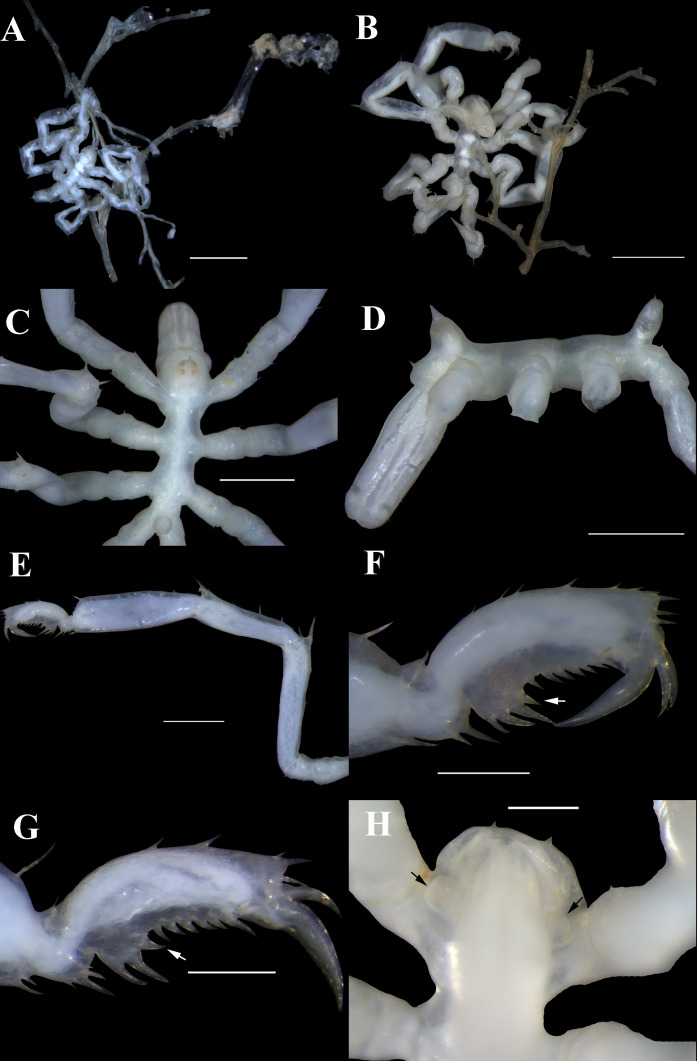
*Endeis straughani*, (TIO2014DY34-5.003): **A**. trunk, dorsal view. (TIO2014DY34-5.002): **B**. trunk, ventral view. (TIO2014DY34-5.001): **C.** trunk, dorsal view; **D.** trunk, lateral view; **E.** leg 1; **F.** tarsus, propodus and claws of leg 1, enlarged, the arrow points to the obliquely short heel spine; **G.** tarsus, propodus and claws of leg 2, enlarged, the arrow points to the obliquely short heel spine. (TIO2014DY34-5.002): **H**. cephalon, ventral view, the arrow points to the oviger bud. Scale bars A and B = 1 mm; C, D and E = 0.5 mm; F, G and H= 0.2 mm.

During the 5th leg of the DY125-34 expedition, which was returning to China from its work zone in the SWIR (Southwest Indian Ocean Ridge), five juvenile *Endeis* specimens were collected along with the hydroid, Leptothecata Cornelius, 1992 while sampling the water for zooplankton. Giving the difficulties associated with identifying juvenile specimens, this small collection (Fig. 1 A, B) was tentatively identified as *Endeis straughani* Clark, 1970 by their compact trunk, robust legs, tubercles located on the femur and on the second tibiae.

## Materials and methods

During the 5th leg (from 5/7/2015 to 5/12/2015) of the DY125-34 expedition, on its return trip to China from the work zone in SWIR, the expedition continued to collect zooplankton samples. These samples were collected by pumping seawater through a pipe which had its intake located 3 m below the surface and the outlet of the pipe was wrapped in a 20 µm mesh net to ensure the minimum size of samples collected. Sampling occurred over a 12-hour period each day. At one point during the return trip, several juvenile specimens of *Endeis* were accidentally collected along with several hydroids. Stock (1968), Clark (1970), Bamber (1979)and Staples (1982)were used to identify the *Endeis* specimens.

The five pycnogonids (No. TIO2014DY34-5.001-005), along with the hydroids, were preserved in 90% ethanol and stored at the Third Institute of Oceanography, Ministry of Natural Resources, China. Photographs were taken with an Auto-montage system on a Leica M205 FA stereomicroscope and z-stacks were created with the LAS software (Version 3.8). Measurements of the pycnogonids were made axially, dorsally for the trunk, laterally for the proboscis and legs and are given in mm.

## Taxon treatments

### Endeis straughani

Clark, 1970

353009C7-CB41-54E1-B160-112DB3BF49E2

Phoxichilus charybdaeus (?). Haswell 1884: 1033.
Endeis straughani Clark 1970: 13-15, figs. 1-5; Krapp 1975: 86 (list); Staples 1982: 461-462 (literature), figs. 5K-M, Pl. 1 figs. A-B; Bamber and Costa 2009: 168 (list), 179 (text).
Endeis picta Bamber 1979: 251-254, figs. 1 A-I.
Phoxichilus charybdaeus (?). Haswell 1884: 1033.
Endeis straughani Clark 1970: 13-15, figs. 1-5; Krapp 1975: 86 (list); Staples 1982: 461-462 (literature), figs. 5K-M, Pl. 1 figs. A-B; Bamber and Costa 2009: 168 (list), 179 (text).
Endeis picta Bamber 1979: 251-254, figs. 1 A-I.

#### Materials

- **Type status:** Other material. **Occurrence:** individualID: TIO2014DY34-5.001; individualCount: 1; lifeStage: juvenile; **Taxon:** scientificName: *Endeis straughani* Clark, 1970; kingdom: Animalia; phylum: Arthropoda; class: Pycnogonida; order: Pantopoda; family: Endeidae; genus: Endeis; **Location:** locationID: Mauritius; verbatimCoordinates: 71.5E; verbatimLatitude: 30.3S; **Identification:** identifiedBy: Jianjia Wang; **Event:** year: 2015; month: 5; day: 7; habitat: amongst hydroids
- **Type status:** Other material. **Occurrence:** individualID: TIO2014DY34-5.002; individualCount: 1; lifeStage: juvenile; **Taxon:** scientificName: *Endeis straughani* Clark, 1970; kingdom: Animalia; phylum: Arthropoda; class: Pycnogonida; order: Pantopoda; family: Endeidae; genus: Endeis; **Location:** locationID: Mauritius; verbatimCoordinates: 71.5E; verbatimLatitude: 30.3S; **Identification:** identifiedBy: Jianjia Wang; **Event:** year: 2015; month: 5; day: 7; habitat: amongst hydroids
- **Type status:** Other material. **Occurrence:** individualID: TIO2014DY34-5.003; individualCount: 1; lifeStage: juvenile; **Taxon:** scientificName: *Endeis straughani* Clark, 1970; kingdom: Animalia; phylum: Arthropoda; class: Pycnogonida; order: Pantopoda; family: Endeidae; genus: Endeis; **Location:** locationID: Mauritius; verbatimCoordinates: 80.4E; verbatimLatitude: 22.3S; **Identification:** identifiedBy: Jianjia Wang; **Event:** year: 2015; month: 5; day: 12; habitat: amongst hydroids
- **Type status:** Other material. **Occurrence:** individualID: TIO2014DY34-5.004; individualCount: 1; lifeStage: juvenile; **Taxon:** scientificName: *Endeis straughani* Clark, 1970; kingdom: Animalia; phylum: Arthropoda; class: Pycnogonida; order: Pantopoda; family: Endeidae; **Location:** locationID: Mauritius; verbatimCoordinates: 80.4E; verbatimLatitude: 22.3S; **Identification:** identifiedBy: Jianjia Wang; **Event:** year: 2015; month: 5; day: 12; habitat: amongst hydroids
- **Type status:** Other material. **Occurrence:** individualID: TIO2014DY34-5.005; individualCount: 1; lifeStage: juvenile; **Taxon:** scientificName: *Endeis straughani* Clark, 1970; kingdom: Animalia; phylum: Arthropoda; class: Pycnogonida; order: Pantopoda; family: Endeidae; genus: Endeis; **Location:** locationID: Mauritius; verbatimCoordinates: 80.4E; verbatimLatitude: 22.3S; **Identification:** identifiedBy: Jianjia Wang; **Event:** year: 2015; month: 5; day: 12; habitat: amongst hydroids

#### Notes

##### Identification

TIO2014DY34-5.001. Trunk glabrous, completely segmented. Collar with flat protuberances laterally. Cephalon with two short spines distally. Lateral processes separated by slightly more than their own diameter, each with 1-3 short distal dorsal spines. Ocular tubercle taller than its base diameter, with pointed peak; two pairs of eyes pigmented distinctly. Abdomen pointing slightly backwards, almost as high as ocular tubercle, with two lateral spines distally (Fig. 1 C, D).

Proboscis barrel-shaped, leaning downwards at about 45 degrees, little longer than half of the trunk, slightly inflated at proximal third, with few short setae along the length and dense short setae around the mouth (Fig. 1 C, D).

Legs slender (Fig. 1 E). First coxa short, second coxa 2 times as long as first or third coxa, with some short setae ventrally. The main articles with setae sparsely dorsally and laterally; femur slightly longer than first tibia, each with one long seta located on the distal tubercle; second tibia longest, with one long setae distally. Tarsus small, subtriangular, with one spine and few setae ventrally. Propodus gently curved, with some short setae dorsally and three long setae distally; heel prominent, with 2-3 robust spines of increasing length distally, with one obliquely short spine (Fig. 1 F, G); sole with 4-6 spines and some slender setae. Main claw stout, half the length of propodus; auxiliary claws slender, almost half of main claw.

*Measurements of TIO2014DY34-5.001* in mm: trunk length from the anterior margin of the cephalon to the tip of 4^th^ lateral processes 1.34; width across second lateral processes 0.68; proboscis length 0.76; abdomen length 0.25. First leg: coxa-1 0.17, coxa-2 0.34, coxa-3 0.18, femur 0.98, tibia-1 0.84, tibia-2 1.04, tarsus 0.06, propodus 0.51, main claw 0.25, auxiliary claw 0.12.

Trunk length (TL) and Trunk Width (TW) of other specimens: TIO2014DY34-5.002 (TL 1.29; TW 0.75), TIO2014DY34-5.003 (TL 1.03; TW 0.58), TIO2014DY34-5.004 (TL 0.99; TW 0.57), TIO2014DY34-5.005 (TL 0.41; TW 0.33).

##### Distribution

The type location for *E. straughani* is Queensland, Australia (Clark 1970). Bamber (1979) described *E. picta* from the coast of Ghana, West Africa which has since been synonymised with *E. straughani* by Staples (1982), thus greatly expanding the previous known range for this species. Our specimens were inadvertently collected from the fouling community on the expedition ship’s hull which probably originated from Port Louis, Mauritius, where the ship had previously been docked, expanding the species range even further.

##### Remarks

Identifying the juvenile *Endeis* specimens was not an easy task, especially since there were no adult males collected, which would have provided information on ovigers and cement glands that are very important for determining species in this genus. Our juvenile specimens agreed generally with the descriptions given by Clark (1970), Bamber (1979) and Staples (1982) and were especially consistent with the sub-adult characteristics mentioned by Staples (1982, p. 462). In addition, lateral process intervals, along with neck and lateral process spines and the variable number of heel spines in a single specimen, a characteristic also present on the adults described in Staples (1982) and which has not been reported for other *Endeis* species, added confirmation that these specimens were *E. straughani* or a very close relative.

**Figure 2.**
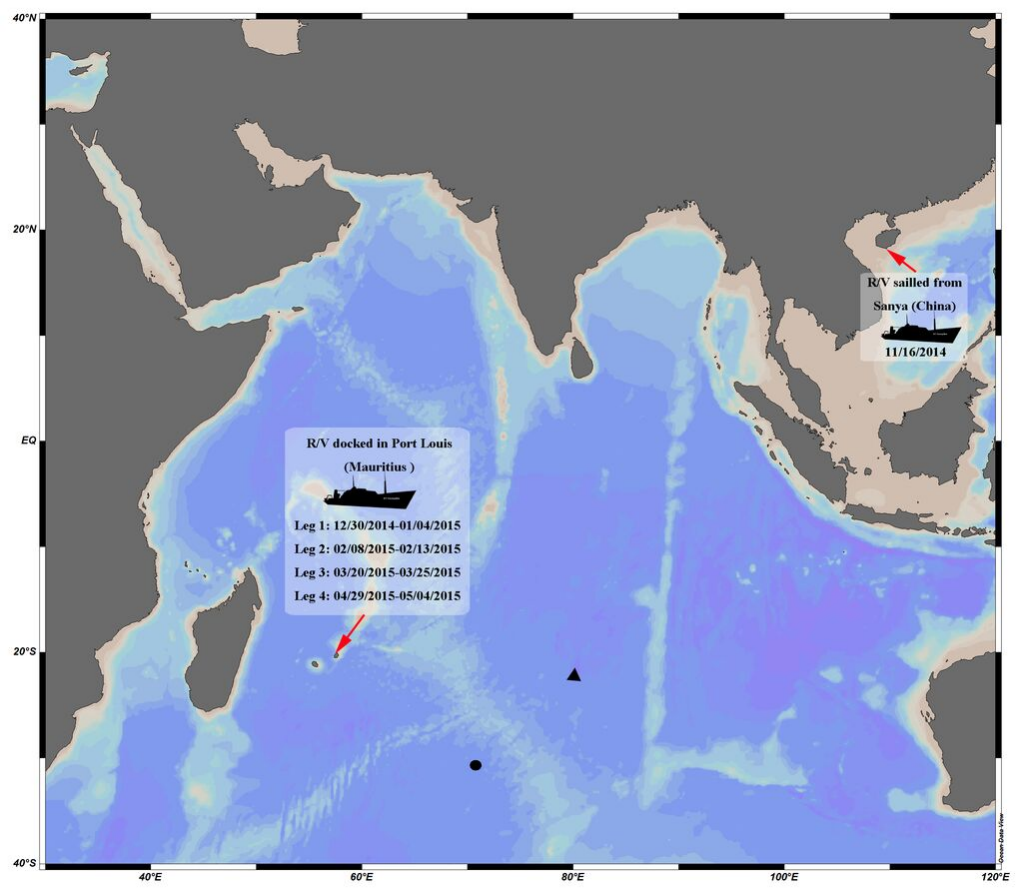
The sampling stations and the docking position and duration of the R/V in the “DY125-34” expedition. ? the sampling location in 05/07/2015; ? the sampling location in 05/12/2015.

Almost nothing is known about the *Endeis* life cycle, other than an incomplete description of the life cycle of *Endeis spinosa* (Montagu, 1808) by Dogiel (1913, Taf. XIX, figs. 1-4 and 7-8), who described the protonymphon larva and several developmental stages in this species. According to the four distinct types of pycnogonid postembryonic development which were characterised by Bain (2003), our *Endeis* specimens probably have the encysted larva type of development, even though we did not examine the hydroids in order to look for the earlier encysted stages.

Three specimens (TIO2014DY34-5.002, TIO2014DY34-5.003 and TIO2014DY34-5.004) have oviger buds (Fig. 1H) and show variations in the length of trunk (range from 0.99 mm to 1.29 mm) and transparency of the cuticle. Of the specimens with oviger buds, which will become adult males, the oldest juvenile (TIO2014DY34-5.002) is larger than the other two individuals and is no longer quite as transparent (Fig. 1B). The largest specimen (TIO2014DY34-5.001) is probably 1 or 2 moults away from the adult stage (Fig. 1C-G).

In the absence of a complete life cycle to compare these specimens and based on the presence of four pairs of legs (4th pair still not completely developed) and also the presence of oviger buds in several of the specimens, they are probably at least 1 or 2 moults away from becoming adults (Dogiel 1913, Bain 2003, Lovely 2005).

*Endeis straughani* can be distinguished from other similar *Endeis* species in the same region (Stock 1965, Stock 1974) as follows: *E. clipeata* (Möbius, 1902) has an apical cylindrical process on the propodus; *E. meridionalis* (Böhm, 1879) has distorted femurs and *E. mollis* (Carpenter, 1904) has widely separated lateral processes and no spurs on the main leg segments. Staples (1982) pointed out the characteristics for distinguishing *E. straughani* from *E. biseriata* Stock, 1968 which has widely separated lateral processes (nearly three times the diameter of a single lateral process).

Stock (1970) recorded two *Endeis* species, *Endeis pauciporosa* Stock, 1970 and *Endeis flaccida* Calman, 1923 from the Gulf of Aqaba and *E. flaccida* also occurred in the Aden Habour (Stock 1968). *Endeis pauciporosa* and *E. flaccida* can be easily distinguished from *E. straughani* since they have distorted femurs and alimentary diverticula in the legs with numerous branched caeca, respectively.

*Endeis viridis* Pushkin, 1976, found in the Crozet Archipelago and Kerguelen Islands (Pushkin 1976, Pushkin 1993), can be distinguished from *E. straughani* by having distinctly slender legs and relatively long auxiliary claws, more than half the length of the main claw.

The specimens sustained some damage, such as unnaturally distorted tibiae and other kinds of damage during collecting, probably due to the pumping actions of the collecting apparatus (Fig. 1 A, B and E).

Clark’s specimens (Clark 1970) retained a bright green colour after being preserved in alcohol and our specimens showed a range from bright purple to nearly transparent after being in alcohol for some time.

## Discussion

Takahashi et al. (2007) indicated that most species of *Endeis* live in shallow waters, although some have been found on floating seaweed. Considering that the intake was fitted with an iron mesh and no seaweed or other attached objects were gathered, we speculate that our specimens were from the fouling communities attached to the ship's hull or to the collecting pipe. The R/V “Dayangyihao” had been defouled before sailing from Sanya (China) and only docked in Port Louis (Mauritius) four times during the expedition (Fig. 2). The hydroids (Order Leptothecata Cornelius, 1992) and the pycnogonids had not previously been recorded from Mauritius before, according to the Mauritius Oceanography Institute (2007) and, if they turn out to be a new species, this will expand the list of sea spiders known from Mauritius.

## Supplementary Material

XML Treatment for Endeis straughani
